# Cortex-driven cytoplasmic flows in elongated cells: fluid mechanics and application to nuclear transport in *Drosophila* embryos

**DOI:** 10.1098/rsif.2023.0428

**Published:** 2023-11-15

**Authors:** Pyae Hein Htet, Eric Lauga

**Affiliations:** Department of Applied Mathematics and Theoretical Physics, University of Cambridge, Cambridge CB3 0WA, UK

**Keywords:** cytoplasmic streaming, *Drosophila*, biological transport, fluid dynamics, intracellular flows

## Abstract

The *Drosophila melanogaster* embryo, an elongated multi-nucleated cell, is a classical model system for eukaryotic development and morphogenesis. Recent work has shown that bulk cytoplasmic flows, driven by cortical contractions along the walls of the embryo, enable the uniform spreading of nuclei along the anterior–posterior axis necessary for proper embryonic development. Here, we propose two mathematical models to characterize cytoplasmic flows driven by tangential cortical contractions in elongated cells. Assuming Newtonian fluid flow at low Reynolds number in a spheroidal cell, we first compute the flow field exactly, thereby bypassing the need for numerical computations. We then apply our results to recent experiments on nuclear transport in cell cycles 4–6 of *Drosophila* embryo development. By fitting the cortical contractions in our model to measurements, we reveal that experimental cortical flows enable near-optimal axial spreading of nuclei. A second mathematical approach, applicable to general elongated cell geometries, exploits a long-wavelength approximation to produce an even simpler solution, with errors below 5% compared with the full model. An application of this long-wavelength result to transport leads to fully analytical solutions for the nuclear concentration that capture the essential physics of the system, including optimal axial spreading of nuclei.

## 1. Introduction

The cell cortex, present in most animal cells, is a thin network of polymeric filaments (actin), molecular motors (myosin) and filament-binding proteins, located directly underneath the cell membrane separating the interior of biological cells from the outside environment [1,2]. The actin polymer network gives the cell its shape and stiffness, while myosin motors exert contractile stresses on actin filaments, enabling the active cortical contractions crucial for the cell’s mechanical and morphological functions [1,2]. The control of cell mechanics underlies numerous biological processes. This includes cell migration via cortical flow-generated propulsive forces [3–6], often aided by the formation of forward protrusions called blebs [7,8]. Similarly, the cortex plays a key role in the shape changes involved in cell division such as mitotic rounding [9] and furrow formation and constriction [10].

An important class of problems in early developmental biology concerns intracellular flows driven by cortical movements [11]. Much of what is known about these cytoplasmic flows (the cytoplasm is the complex fluid filling the inside of biological cells) has been obtained from work on model organisms, including the fruit fly *Drosophila melanogaster*, the nematode *Caenorhabditis elegans* and the zebrafish *Danio rerio*. This type of system belongs to a broader class of ‘cytoplasmic streaming’ problems, where large-scale flows are induced in large eukaryotic cells, including algae, plants, amoeba, fungi and—of particular interest in this paper—animal cells in early development [12–14].

Waves of actin polymerization along the cortex of the zebrafish zygote (i.e. fertilized egg) create the flows responsible for cytoplasmic transport of yolk granules [15,16]. In *C. elegans* zygotes, actin–myosin contractions play a crucial role in cell polarization (i.e. the creation of asymmetry in cellular organization) [17]. The cortex flows towards the anterior pole and thus creates cytoplasmic flows along the cell axis directed towards the posterior end, distributing cortical and cytoplasmic cellular components asymmetrically in the anterior–posterior (AP) direction.

**Figure 1.**
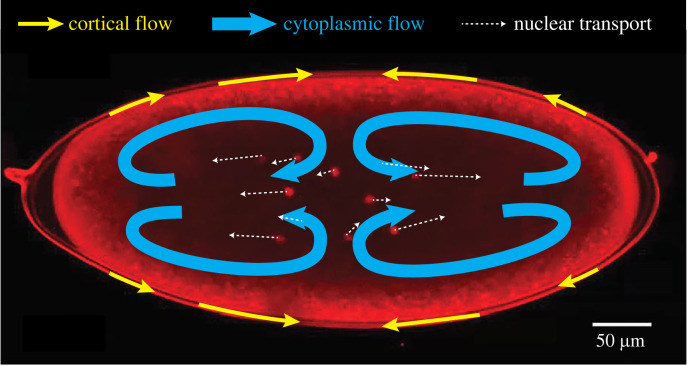
An experimental image of cytoplasmic transport in *Drosophila* embryo, reprinted from Deneke *et al.* [18], © (2019), with permission from Elsevier (Supplementary video 3). Red dots located near the centre are nuclei produced by cell division. Arrows are overlaid onto the image to schematically indicate cortical flows at the edge of the cell (thin yellow arrows), cytoplasmic flow inside the embryo (thick blue arrows) and the resultant transport of nuclei (thin dotted arrows).

In this paper, we consider the specific case of *Drosophila* embryos, which are ‘syncytial’, meaning that the numerous nuclei obtained in the early few cycles of cell division are not separated into individual cells but instead share the same cytoplasm. In early development of the embryo, the cortex contracts towards the centre of the cell, most prominently in cell cycles 4–6 (cell cycle *n* refers to the *n*th cycle of cell division), thereby driving cytoplasmic flows that spread the daughter nuclei along the AP axis of the embryo, ensuring a uniform positioning of nuclei across the embryo [18]. This process is illustrated in figure 1 (reproduced from Supplementary video 3 in [18]) showing the cortical flows at the edge of the cell (thin yellow arrows), and the resulting cytoplasmic flow inside the embryo (thick blue arrows), which then transports the nuclei generated by cell division (thin dotted arrows).

The rich mechanobiology of the cell cortex has attracted interest from the physics and mathematics community, spurring the development of active gel theories in recent years, which describe the cortex as a thin layer of viscoelastic material with active tension [19–23]. Such a framework may be coupled with a model for the flow to reproduce the full dynamics of the surrounding fluid. Recent work couples active gel theory with a computational method for the flow (the immersed boundary method) to investigate how a cell deforms in an external flow and simulate cytoplasmic flows generated by cortical movement [24].

While these methods are able to model cortical dynamics in great detail, solving the active gel equations is often a complicated and computationally intensive process [23]. The real biological system is very complex, consisting of an active cortical actin–myosin mesh coupled mechanically and biochemically to the cytoplasm and its components, and modelling all these details faithfully is inevitably a task that requires a computational approach [25].

Biophysical insight could, however, be obtained from simplified models amenable to analytical treatment. In particular, despite its complex chemical composition, the cytoplasm can be described as an effective Newtonian fluid over time scales of interest, a modelling assumption supported by past works comparing flow computations with particle image velocimetry (PIV) data [18,26,27] and by rheological measurements [28], while the forcing from the cortex may be modelled as an effective boundary slip velocity [18,27]. Naturally, such a simple approach has its limitations, and is not able to explain all quantitative features of the cytoplasmic flow. For instance, the locations of the vorticity extrema in the experimentally measured flow in the *Drosophila* embryo and the required cortical flow velocities deviate from the predictions from a simple Stokes flow (i.e. a Newtonian fluid flow in the absence of inertia), and a more detailed two-fluid model has been proposed to rectify these disparities [25]. Nonetheless, a Stokes flow model does reproduce well the large-scale features of the flow [18] and its mathematical simplicity enables further analytical development.

For axisymmetric incompressible flow in a spherical geometry, the Stokes equations may be solved exactly using a series expansion of the streamfunction [29]. This general solution has been used to study the axisymmetric flow induced inside a spherical shell by small radial deformations of the boundary, as a model for the cytoplasmic flows generated in starfish oocytes by cortical contractions [26].

However, often the relevant biological cells are elongated, as in the case of *Drosophila* embryos and *C. elegans* zygotes, introducing an additional level of geometrical complexity to simplified spherical models. Motivated by the problem of cortical contractions in syncytial *Drosophila* embryos, and the resulting flow-based transport of nuclei [18], we propose in this paper two analytical mathematical methods to model the cytoplasmic flows generated inside an elongated cell created by arbitrary axisymmetric cortical flow.

Our first approach consists of solving exactly for the Stokes flow in a spheroidal model cell driven at the boundary via a slip velocity. We then show how this three-dimensional flow solution can be applied to transport in the *Drosophila* embryo to obtain new insight on intracellular transport. The second method, valid for any elongated shape, uses a long-wavelength approximation to produce an even simpler, yet remarkably accurate, lubrication solution for the cytoplasmic flows. We exploit this result to construct, and analytically solve, a reduced model of nuclear transport capturing all the main features of nuclear spreading along the embryo axis.

Our paper is organized as follows. We first construct the simplest possible Stokes flow model able to capture the relevant physics of the problem. We obtain analytical solutions for the bulk flow resulting from cortical contractions on the cell boundary without the need for a numerical approach (§2). We then use our solution to reveal that the cortical flows in the *Drosophila* embryo are finely tuned to ensure the optimal spreading of nuclei along the AP axis (§3). We next derive a long-wavelength solution applicable to any elongated shape (§4). Finally, we use the long-wavelength flow to analytically solve a one-dimensional transport model which reproduces all important features of the full mathematical model (§5).

## 2. Model for cytoplasmic streaming in elongated cells: boundary-driven Stokes flows in a prolate spheroid

In order to capture cytoplasmic streaming in elongated cells, we propose a first mathematical model where the cell is represented by a prolate spheroid and the flow, assumed to be Newtonian and in the Stokes regime, is driven from the boundary. After detailing the specific problem statement (§2.1), we introduce prolate spheroidal coordinates (§2.2) and the streamfunction in these coordinates (§2.3). We solve for the flow analytically using a series solution for the streamfunction (§2.4) and illustrate the first few modes of the resulting flow field in terms of the appropriate basis function decomposition of the prescribed tangential slip velocities (§2.5).

### 2.1. Problem statement

**Figure 2.**
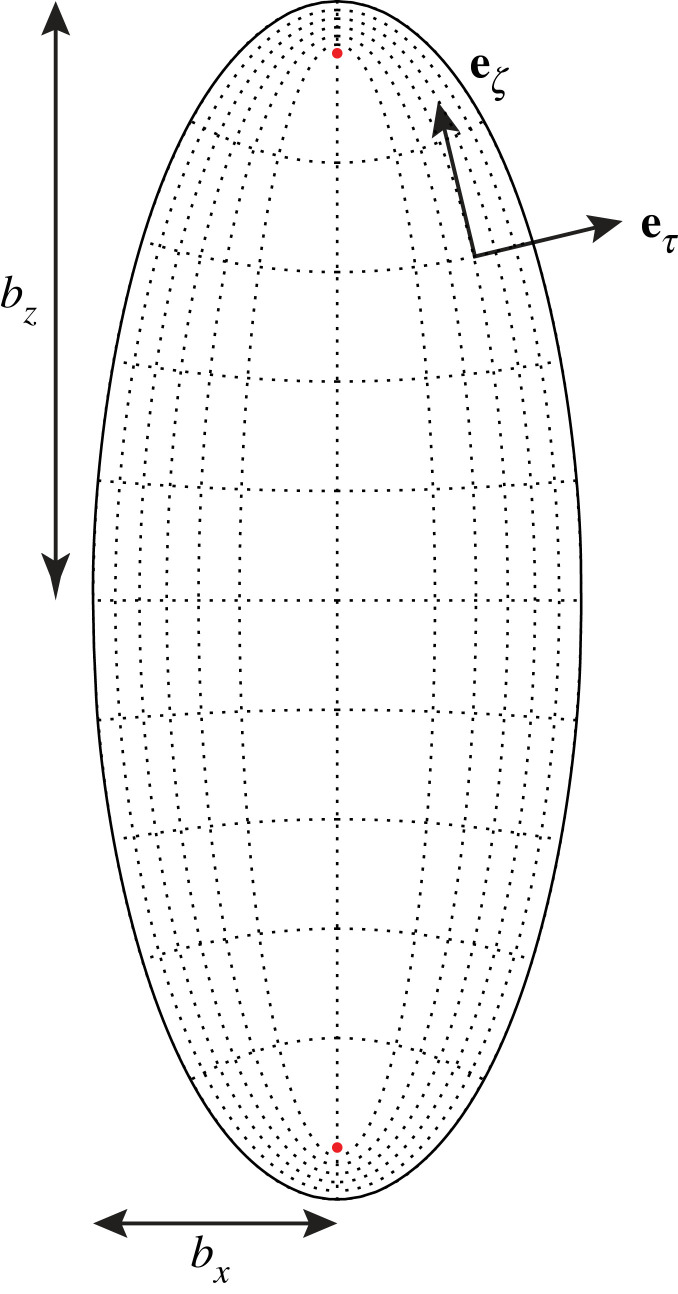
Cross-section containing the long axis of a prolate spheroid of semi-major axis *b*_*z*_ and semi-minor axis *b*_*x*_, illustrating the modified prolate spheroidal coordinate system (*τ*, *ζ*, *ϕ*) and the basis vectors $e_{\tau }$ and $e_{\zeta }$. Since the flow is axisymmetric, the azimuthal angle *ϕ* is omitted from illustration. Dotted lines are isosurfaces of *τ* and *ζ*. The red dots are the foci at *z* = ±*c*.

We consider an incompressible Newtonian fluid, of dynamic viscosity *μ*, inside a rigid prolate spheroidal domain oriented along the *z*-axis, of semi-major axis *b*_*z*_ and semi-minor axis *b*_*x*_ < *b*_*z*_ (see sketch in figure 2). The flow field **u** inside the spheroid is driven by a prescribed axisymmetric tangential slip velocity **v**_*s*_ along the boundary. We assume that the relevant velocity scale *U* and length scale *L* are sufficiently small such that we may neglect inertia, i.e. the Reynolds number *Re* = *ρUL*/*μ* ≪ 1 (where *ρ* is the mass density of the fluid). The fluid flow is then governed by the incompressible Stokes equations

2.1 $$\mu \nabla^{2}u=\nabla p,\;\nabla \cdot u=0,$$

where *p* is the pressure field, subject to the no-slip boundary condition

2.2 $$u=v_{s},$$

on the surface of the spheroid.

### 2.2. Coordinate system

We introduce a prolate spheroidal coordinate system [29] (*η*, *θ*, *ϕ*) with semi-focal distance $c\;:=\sqrt{b_{z}^{2}-b_{x}^{2}}$, related to the cylindrical polar coordinates (*r*, *z*, *ϕ*) via

2.3 $$\begin{array}{cc} & r=c\sinh\eta \sin\theta \\ \mathrm{and}\;\;\; & z=c\cosh\eta \cos\theta \end{array}\}$$

and further make the transformation

2.4 τ=cosh⁡ηandζ=cos⁡θ.

In these modified prolate spheroidal coordinates [29], the boundary of the domain is given by

2.5 $$\tau =\tau_{0}\;:=\frac{b_{z}}{c},$$

and the interior of the domain corresponds to 1 ≤ *τ* < *τ*_0_ [30]. Surfaces of constant *τ* are confocal prolate spheroids, with the degenerate spheroid *τ* = 1 being the line segment connecting the foci *z* = ±*c*. The basis vectors (**e**_*τ*_, **e**_*ζ*_) in these modified prolate spheroidal coordinates are related to cylindrical polar coordinates by

2.6 $$e_{\tau }=\frac{\sqrt{\tau^{2}-1}}{\sqrt{\tau^{2}-\zeta^{2}}}\left(\frac{\tau \sqrt{1-\zeta^{2}}}{\sqrt{\tau^{2}-1}}e_{r}+\zeta e_{z}\right)$$

and

2.7 $$e_{\zeta }=\frac{\sqrt{1-\zeta^{2}}}{\sqrt{\tau^{2}-\zeta^{2}}}\left(\frac{-\zeta \sqrt{\tau^{2}-1}}{\sqrt{1-\zeta^{2}}}e_{r}+\tau e_{z}\right).$$

The Lamé metric coefficients $h_{\phi },h_{\zeta }$ and *h*_*τ*_ associated with the prolate spheroidal coordinates are given by

2.8 $$h_{\phi }=c\sqrt{\tau^{2}-1}\sqrt{1-\zeta^{2}},$$

2.9 $$h_{\zeta }=c\frac{\sqrt{\tau^{2}-\zeta^{2}}}{\sqrt{1-\zeta^{2}}}$$

2.10 $$\mathrm{and}\;\;\;\;h_{\tau }=c\frac{\sqrt{\tau^{2}-\zeta^{2}}}{\sqrt{\tau^{2}-1}}.$$

The set-up and coordinate system are illustrated in figure 2.

### 2.3. Streamfunction

Exploiting the axisymmetry of the problem, we seek a solution in terms of a Stokes streamfunction *ψ*(*τ*, *ζ*) satisfying $u=\nabla \times (\psi e_{\phi }/h_{\phi })$ [29], or more explicitly,

2.11 $$u=u_{\tau }e_{\tau }+u_{\zeta }e_{\zeta }=\frac{1}{h_{\zeta }h_{\phi }}\frac{\partial \psi }{\partial \zeta }e_{\tau }-\frac{1}{h_{\tau }h_{\phi }}\frac{\partial \psi }{\partial \tau }e_{\zeta }.$$

Note that a streamfunction formulation automatically ensures incompressibility, ∇⋅u=0.

Writing the tangential slip velocity at the boundary as $v_{s}=v_{s}e_{\zeta }$, we have two boundary conditions for the streamfunction: (i) impenetrability,

2.12 $$u_{\tau }(\tau_{0},\zeta )=0,$$

and (ii) the prescribed slip velocity,

2.13 $$u_{\zeta }(\tau_{0},\zeta )=v_{s}(\zeta ).$$

### 2.4. Solution

The general ‘semiseparable’ solution for the streamfunction was derived by Dassios *et al.* [31]. Recently, Pöhnl *et al.* [30] used this general solution to solve for the swimming velocity of, and the flow field outside, a spheroidal squirmer with an axisymmetric tangential slip velocity. In the first mathematical part of the current paper, we adapt this approach to solve the complementary ‘interior’ problem.

The general solution for the streamfunction is given by

2.14 $$\psi (\tau ,\zeta )=g_{0}(\tau )G_{0}(\zeta )+g_{1}(\tau )G_{1}(\zeta )+\sum_{n=2}^{\infty }[g_{n}(\tau )G_{n}(\zeta )+h_{n}(\tau )H_{n}(\zeta )],$$

where *G*_*n*_ and *H*_*n*_ are Gegenbauer functions of the first and second kind, respectively [32], and *g*_*n*_ and *h*_*n*_ are specific linear combinations of *G*_*n*_ and *H*_*n*_.

Now the *H*_*n*_s are singular at *ζ* = ±1 (i.e. along the *z*-axis, between the poles and the foci), thus requiring *h*_*n*_ = 0 for all *n*. *G*_0_(*ζ*) = 1 and *G*_1_(*ζ*) = −*ζ* are non-zero at *ζ* = ±1 so we also require *g*_0_ = *g*_1_ = 0; otherwise $v_{\zeta }$ would diverge at *ζ* = ±1 because of the Lamé metric coefficients. Non-zero *g*_*n*_s are allowed for *n* ≥ 2, however, since *G*_*n*≥2_( ± 1) = 0. The general solution from equation (2.14), therefore, reduces to

2.15 $$\psi (\tau ,\zeta )=\sum_{n\geq 2}g_{n}(\tau )G_{n}(\zeta ).$$

Upon further using a similar condition that the *G*_0_(*τ*) and *G*_1_(*τ*) terms cannot be present since the solution would otherwise diverge at *τ* = 1, the admissible terms in the *g*_*n*_s are

2.16a $$g_{2}(\tau )=F_{2}G_{2}(\tau )+E_{4}G_{4}(\tau ),$$

2.16b $$g_{3}(\tau )=F_{3}G_{3}(\tau )+E_{5}G_{5}(\tau )$$

2.16c $$\begin{array}{r} \mathrm{and}\;g_{n\geq 4}(\tau )=F_{n}G_{n}(\tau )+E_{n+2}G_{n+2}(\tau )+E_{n}G_{n-2}(\tau ), \end{array}$$

where {*E*_*n*_, *F*_*n*_} are constants to be determined from the boundary conditions.

#### 2.4.1. Boundary conditions for $u_{\tau }$

The impenetrability condition implies

2.17 $$\frac{\partial \psi }{\partial \zeta }(\tau_{0},\zeta )=0,$$

i.e. *ψ* is constant on the boundary. Since *ψ* is zero at the poles (*ψ*(*τ*_0_, *ζ*) = 0) from the property *G*_*n*≥2_(*ζ* = ±1) = 0 of the Gegenbauer functions, *ψ* is zero everywhere on the boundary, yielding our first boundary condition for the *g*_*n*_s,

2.18 $$g_{n}(\tau_{0})=0.$$

#### 2.4.2. Boundary conditions for $u_{\zeta }$

We write the tangential velocity boundary condition in terms of *ψ* and use the orthogonality relations

2.19 $$\begin{array}{r} \int_{-1}^{1}\frac{G_{n}(\zeta )G_{m}(\zeta )}{1-\zeta^{2}}\;d\zeta =\frac{2}{n(n-1)(2n-1)}\delta_{mn},\;n,m\geq 2, \end{array}$$

and the relation ${\left(1-\zeta^{2}\right)}^{1/2}P_{l}^{1}(\zeta )=-l(l+1)G_{l+1}(\zeta )$ between the Gegenbauer functions and the associated Legendre polynomials $P_{l}^{1}$ to obtain

2.20 $$\frac{dg_{n}}{d\tau }(\tau_{0})=c^{2}\int_{-1}^{1}\sqrt{\tau_{0}^{2}-\zeta^{2}}P_{n-1}^{1}(\zeta )v_{s}(\zeta )\;d\zeta .$$

This motivates the expansion of the prescribed boundary flow *v*_*s*_ as

2.21 $$v_{s}(\zeta )=\frac{\tau_{0}}{\sqrt{\tau_{0}^{2}-\zeta^{2}}}\sum_{n\geq 1}B_{n}P_{n}^{1}(\zeta ).$$

The tangential velocity boundary condition may then be expressed as

2.22 $$\frac{dg_{n}}{d\tau }(\tau_{0})=\tau_{0}c^{2}n(n-1)B_{n-1}.$$

The coefficients *B*_*n*_ are determined by the prescribed boundary flow *v*_*s*_, and explicit expressions follow from equation (2.21),

2.23 $$B_{n}=\frac{n+\frac{1}{2}}{n(n+1)\tau_{0}}\int_{-1}^{1}v_{s}(\zeta )\sqrt{\tau_{0}^{2}-\zeta^{2}}P_{n}^{1}(\zeta )\;d\zeta .$$

Note that on the boundary *τ* = *τ*_0_ of the spheroid, *ζ* is simply a rescaled axial position, i.e. *ζ* = *z*/*b*_*z*_.

#### 2.4.3. Solving the boundary conditions for {*E*_*n*_, *F*_*n*_}

As is standard in many Stokes flow problems [29], these boundary conditions yield an infinite system of linear equations for an infinite number of unknowns {*E*_*n*_, *F*_*n*_}, which we may solve order by order and thereby, from equations (2.11), (2.15) and (2.16), determine the full flow field inside the spheroid.

We solve the two linear equations from the boundary conditions, equations (2.18) and (2.22), for *n* = 2 together with equation (2.16*a*) for *g*_2_ for the two unknowns *F*_2_ and *E*_4_. At *n* = 4, we similarly have two equations involving {*E*_4_, *E*_6_, *F*_4_}, which we may again solve since *E*_4_ is known from the previous set of equations; this procedure may be continued to determine {*E*_*n*_, *F*_*n*_} for all even *n*. An analogous method determines {*E*_*n*_, *F*_*n*_} for odd *n*, and we may thus calculate the coefficients to arbitrary order.

### 2.5. Flow fields

In the rest of this paper, we truncate our solution at *n* = 14, since higher orders give minimal gains in accuracy; making instead, for instance, the choice *n* = 25 leads to a difference of 0.1% or less (except at points where the flow is already almost zero).

**Figure 3.**
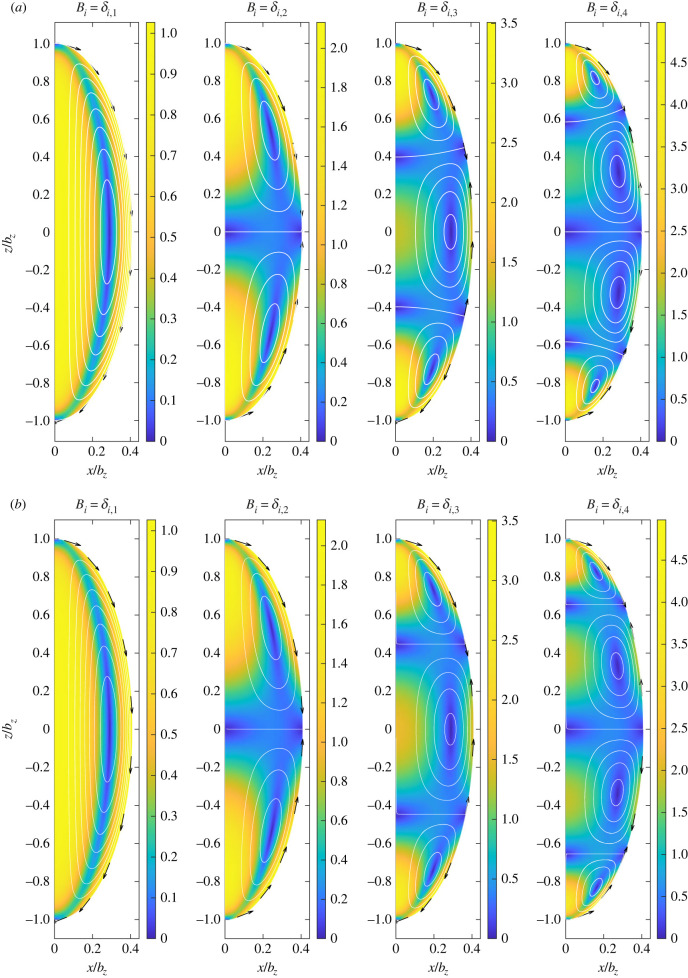
(*a*) Flow fields corresponding to the first four modes in the series expansion in equation (2.21) of the tangential slip velocities using the exact solution from §2. Colour indicates the magnitude |**u**(**x**)| of the velocity field. White lines are streamlines. Black arrows at the boundary indicate tangential velocities. (*b*) Same result as derived by the long-wavelength model of §4.

We now consider the geometry relevant to *Drosophila* embryos, with *b*_*x*_ = 110 μm and *b*_*z*_ = 270 μm, corresponding to *τ*_0_ = 1.095 [18]. We show in figure 3, the flow fields corresponding to the first four modes, i.e. the cases with *B*_*i*_ = *δ*_*im*_ for *m* = 1, 2, 3, 4. The flow field corresponding to the *m*th mode is seen to display *m* vortices inside the cell.

## 3. Optimal nuclear transport by cortex-driven cytoplasmic flows in *Drosophila* embryo

We now use the full flow solution we have computed to study the specific features of flows and transport in the *Drosophila* embryo. After fertilization, the nucleus of the *Drosophila* embryo undergoes 13 rounds of cell division, referred to as ‘cell cycles’ [33,34]. During the first three cell cycles, the nuclei produced by cell division undergo very little movement. However, during cell cycles 4–6, the nuclei are seen experimentally to spread along the long axis of the embryo [35]. This so-called ‘nuclear spreading’ is driven by cortical contractions and the resulting cytoplasmic flows [18]. Once the nuclei have achieved a uniform distribution along the AP axis, from cell cycle 7 onwards, cytoplasmic flows and nuclear movements become significantly smaller. In this section, we use our mathematical solution to investigate the flow-based nuclear spreading in cell cycles 4–6. Specifically, we show theoretically how our mathematical model can reveal near-optimal cortical flows for nuclear spreading.

### 3.1. Cytoplasmic flows during cell cycles 4–6

**Figure 4.**
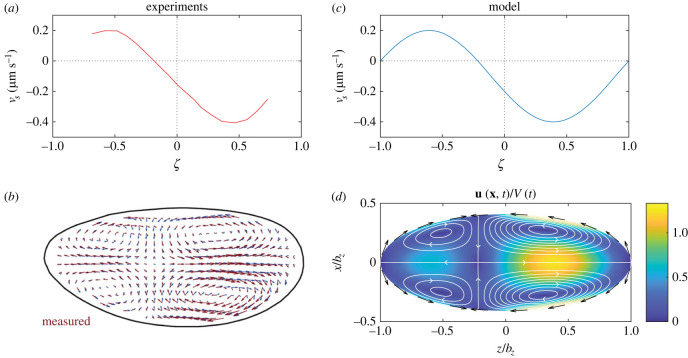
Modelling cytoplasmic flows during cell cycles 4–6 of *Drosophila* development. (*a*,*b*) Experimental measurements of (*a*) the cortical flow profile at the contraction peak of cell cycle 6, redrawn from Deneke *et al.* [18] and (*b*) cytoplasmic flows inside the *Drosophila* embryo (red arrows), reprinted from Deneke *et al.* [18], © (2019), with permission from Elsevier. (*c*) The cortical flow *v*_*s*_ as prescribed in the mathematical model, equation (3.1). (*d*) The cytoplasmic flows obtained from the analytical solution. Black arrows indicate cortical flows at the boundary, streamlines are illustrated in white and the colour map indicates flow speed.

The *Drosophila* embryo is approximately a prolate spheroid with semi-minor axis *b*_*x*_ = 110 μm and semi-major axis *b*_*z*_ = 270 μm (see figures 1 and 4*b*)[18]. Mapping onto our modified prolate spheroidal coordinates, these dimensions correspond to $c=\sqrt{b_{z}^{2}-b_{x}^{2}}=246.6\;\mu m$ and *τ*_0_ = *b*_*z*_/*c* = 1.095.

To model the cortical flow in space and time, we use the experimentally measured cortical flows in cell cycle 6 of the *Drosophila* embryo [18], reproduced in figure 4*a*, with full flow field shown in figure 4*b*. We model this flow distribution with a shifted sine profile; this approximates the experimental profile well in the range of *ζ* in which measurements are taken, and extrapolates to zero at the poles, which we would expect since there are no sources/sinks of cortical matter at the poles. Real-life cortical flows have a complex space- and time-dependence, but to a good approximation may be modelled with a time-dependent amplitude *V*(*t*) ≥ 0 and a spatial profile independent of time, given by the fitted shifted sine function. The boundary conditions for the flows are, therefore, given by

3.1 $$v_{s}(\zeta ,t)=V(t)\left[-\sin\left(\pi \zeta +\arcsin\frac{1}{3}\right)-\frac{1}{3}\right].$$

This boundary profile, with *V*(*t*) taken to be 0.3 μm s^−1^ at the cortical contraction peak of cell cycle 6, is illustrated in figure 4*c*, closely matching the experimental profile from figure 4*a*.

Using equation (3.1) as boundary condition, the flow inside the spheroid is computed as outlined in §2. First the *B*_*n*_s are evaluated using equation (2.23). Next the boundary condition in equations (2.18) and (2.22), with the *g*_*n*_s as defined in equation (2.16), are solved for the coefficients {*E*_*n*_, *F*_*n*_} as explained in section §2.4.3. This determines the streamfunction (equation (2.15)) and in turn the flow field (equation (2.11)).

The experimentally measured flow field is shown as a vector field in figure 4*b*, and we plot our theoretical prediction in figure 4*d*. The analytical model matches experiments well, capturing the cortical flows directed towards the middle of the embryo that then drive cytoplasmic flows along the long axis towards the poles. The model also reproduces the fore–aft asymmetry of the flow (resulting from the asymmetric boundary flows evident in figure 4*a*,*c*) and the four-vortex structure of the bulk flow.

### 3.2. Nuclear transport

#### 3.2.1. Modelling

One purpose of the cytoplasmic flows shown in figure 4 in cell cycles 4–6 is to distribute the cell nuclei along the AP axis of the embryo. These nuclei are subject to both advective transport from the cytoplasmic flows and to Brownian motion.

First, let us show that Brownian motion can be neglected. Each nucleus produced by cell division has a diameter of approximately 7 μm. Assuming a cytoplasm at room temperature with an effective viscosity equal to that of water, a simple application of the Stokes–Einstein relationship allows us to estimate the diffusion constant of each nucleus as approximately $0.06\;\mu m^{2}\;s^{-1}$. Over the course of cell cycles 4–6 (roughly 30 min), the contribution from diffusion to the root mean squared displacement of a nucleus is thus estimated to be at most of the order of 10 μm, much smaller than the typical size of the embryo. Further, the true diffusive displacement is likely to be significantly smaller, because the cytoplasm is known to be more viscous than water [36] and because confinement effects within the *Drosophila* embryo decrease the Stokes mobility of the nuclei [37]. It is thus appropriate to assume that the nuclei do not diffuse appreciably over the time scale of cell cycles, and we may neglect Brownian diffusion in their transport during cycles 4–6.

In the beginning of cell cycle 4, a cloud of nuclei of radius approximately 60 μm is found within the anterior half of the embryo, and is subsequently spread along the long axis [18,38]. In order to model transport, we simulate the advection of a cloud of *N* passive tracer particles drawn from a uniform distribution over a sphere of radius 60 μm centred at the stagnation point of the cytoplasmic flows, i.e. at the point ζ=−2arcsin⁡(1/3)/π≈−0.2163 on the long axis (figure 4*a*). The particles are then simply advected by the cytoplasmic flow, **u**. Specifically, the rate of change of the position **x**_*i*_ of the *i*th particle is equal to the instantaneous flow **u**(**x**_*i*_, *t*) at position **x**_*i*_ and time *t*, i.e. it satisfies the differential equation

3.2 $$\frac{dx_{i}}{dt}=u(x_{i},t).$$

#### 3.2.2. Integral measure of cortical contractions

Under these modelling assumptions, nuclear transport inside the embryo can be shown to depend on a single integral measure of the cortical contractions. Indeed, Stokes flows (i.e. flows of Newtonian fluids in the absence of inertia) have no dependence on time other than through the boundary conditions [37]. Since the advection equation for the nuclei, equation (3.2), is deterministic, the state of the system is fully determined by the quantity *χ* defined as the integral in time of the amplitude of cortical contractions *V*(*t*) from equation (3.1), i.e.

3.3 $$\chi \;:=\int_{0}^{t}V(t^{\prime })\;dt^{\prime }.$$

This quantity *χ* has units of distance, and can be interpreted as (approximately) the total distance travelled by material points along the embryo boundary; it can, therefore, be thought of as a rescaled time, where *χ* increases with time (provided that *V*(*t*) is positive), with the precise details of *V*(*t*) no longer important. In the following simulations, we, therefore, use *χ* rather than *t* as the time variable.

**Figure 5.**
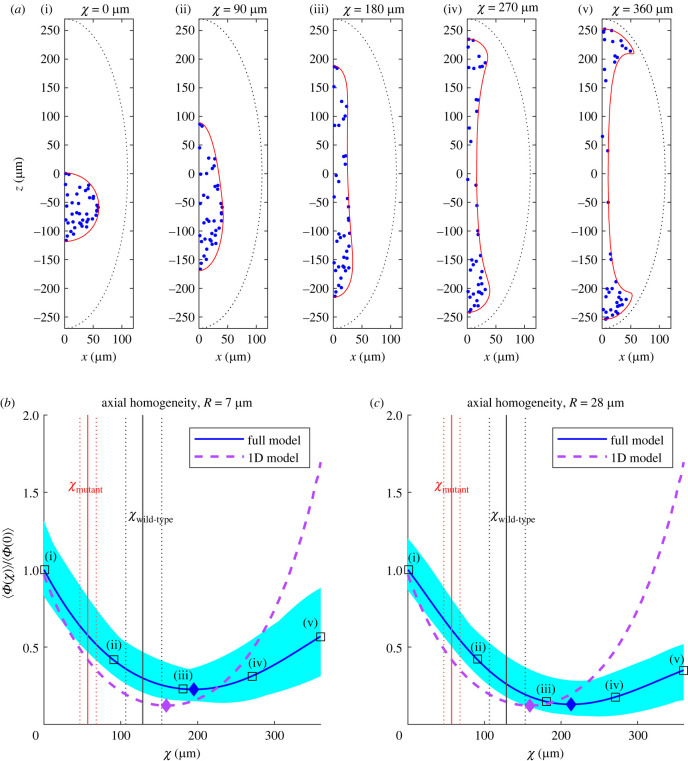
Optimal spreading of nuclei by cortical contractions. (*a*) Advective transport of a cloud of tracer particles (blue) as models for nuclei initially at random positions in a sphere of radius 60 μm, at different values of the rescaled time *χ*, using the exact flow solution from §3.1. Advection of the sphere’s boundary is illustrated in red. (*b*,*c*) Blue solid line: measure of axial homogeneity against *χ*, obtained by computing the mean variance, ⟨Φ(χ)⟩, averaged over 100 simulations of nuclear transport, relative to its mean initial value ⟨Φ(0)⟩, using the density kernel length scales *R* = 7 μm (*b*) and *R* = 28 μm (*c*); shaded area indicates the minimum and maximum value of Φ at each *χ*. Black open squares labelled (i)–(v) correspond to the values of *χ* in the snapshots in (*a*). Blue diamondindicates the optimal value of *χ* (which minimizes ⟨Φ(χ)⟩). Black and red lines indicate estimates of the average (solid lines) value of *χ* realized in wild-type and mutant (PPI-het) *Drosophila* embryos, respectively, and at the 95% confidence levels (dotted lines). The purple dashed line indicates axial homogeneity as predicted by a reduced one-dimensional model of transport (§5), with the purple diamond indicating the optimal *χ* as predicted by that reduced model.

The simulation results are shown in figure 5*a* in the case *N* = 40, and illustrate how the nuclei, initially arranged within a 60 μm sphere, are progressively spread by cytoplasmic flows along the axis of the embryo as the value of *χ* increases. We further plot in red how the boundary of the initial sphere is distorted by the flows. Strikingly, we see from these computational results that the bulk flows (driven by cortical contractions) initially facilitate the axial spreading of the nuclei; however, beyond some critical value of *χ*, applying flows further appears to be counterproductive, as they produce a ‘neck’ scarce in particles and accumulate a disproportionate number of nuclei near the poles of the embryo, eventually resulting in recirculation of nuclei towards the centre along the boundary.

#### 3.2.3. Optimal cytoplasmic transport and cortical contractions

These numerical results, therefore, seem to indicate that an optimum *χ* exists to ensure a uniform axial spreading of the nuclei. To make this observation more quantitative, we introduce a mathematical measure of axial homogeneity in the distribution of nuclei. Specifically, we define a continuous density function *ρ*(*z*, *χ*) of nuclei along the long *z*-axis of the embryo by placing a Gaussian kernel at the centre of each nucleus. With *N* nuclei, we define

3.4 $$\rho (z,\chi )\;:\;=\frac{1}{NR\sqrt{2\pi }}\sum_{i=1}^{N}e^{-{\left[z-z_{i}(\chi )\right]}^{2}/2R^{2}},$$

where *z*_*i*_(*χ*) is the position of the *i*th nucleus along the long axis at rescaled time *χ* and where the length scale *R* controls the width of the Gaussian kernel. The homogeneity of particle distribution along the axis may then be measured by the variance Φ of the density, defined as

3.5 $$\Phi (\chi )\;:=\int_{-b_{z}}^{b_{z}}{\left[\rho (z,\chi )-\bar{\rho }(\chi )\right]}^{2}\;dz,$$

where $\bar{\rho }(\chi )=(1/2b_{z})\int_{-b_{z}}^{b_{z}}\rho (z,\chi )\;dz$ is the average density; note that the variance is only a function of *χ*.

With these definitions, we run 100 simulations (i.e. 100 sets of random initial conditions for the model nuclei) and plot the mean value ⟨Φ(χ)⟩, relative to the average initial value in the simulations ⟨Φ(0)⟩, for two biologically relevant values for the Gaussian kernel length scale: (i) *R* = 7 μm, which is the approximate diameter of a nucleus (results shown in figure 5*b*, solid line) and (ii) *R* = 28 μm, the mean nuclear separation distance determined by microtobule aster migration (an aster is a radial array of microtubules attached to a centrosome) during cell division [39] (results shown in figure 5*c*). In each plot, the shaded regions indicate the minimum and maximum over the 100 simulations of Φ(χ) at each *χ*. These quantitative results confirm the intuitive observation gleaned from the images in figure 5*a*: the optimal axial homogenization of the nuclei happens for a well-defined value of the integral measure of cortical contractions, *χ*. Specifically, this optimal spreading is predicted by our numerical simulations to take place at *χ* ≈ 195 μm using *R* = 7 μm and *χ* ≈ 213 μm using *R* = 28 μm. (Here, and in what follows, we give all figures for *χ* to the nearest integer value.)

**Figure 6.**
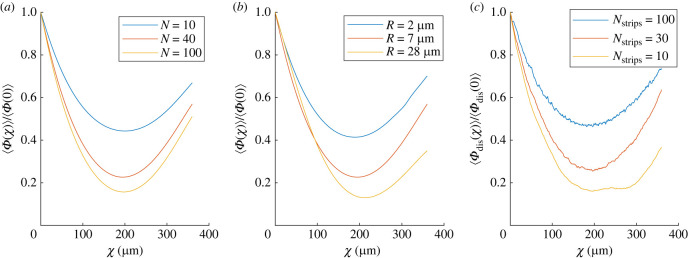
Dependence of optimal cortical forcing on modelling parameters. (*a*) Average axial homogeneity, ⟨Φ(χ)⟩, with *R* = 7 μm, plotted for three different numbers *N* of particles advected by the flow. (*b*) Average axial homogeneity, ⟨Φ(χ)⟩, for *N* = 40 particles, plotted for three different values of the Gaussian kernel length scale, *R*. (*c*) Alternative approach where a different, discrete, measure of axial homogeneity is introduced, $\left\langle \Phi_{\mathrm{dis}}(\chi )\right\rangle$ (see text), with results shown for *N* = 40 particles and three values of the number *N*_strips_ of strips.

Importantly, the presence of this optimal spreading is very robust to changes in our modelling parameters. Varying the number of particles *N* advected by the flow produces essentially no difference, as illustrated in figure 6*a* where the result for *R* = 7 μm and *N* = 40 (optimum at *χ* ≈ 195 μm) is compared with those for *N* = 10 and 100 (optima at *χ* ≈ 200 and 197 μm, respectively). Changing the length scale *R* associated with the Gaussian kernel in the definition of the continuous density, equation (3.4), also has no qualitative effect on our results, as illustrated in figure 6*b*, and the optimal value of *χ* is seen to only increase weakly with *R*: our model predicts optima at χ≈189,195 and 213 μm for the choices *R* = 2, 7 and 28 μm, respectively.

Note that alternatively, we could use a ‘discrete’ approach to quantify axial homogeneity. To do this, we divide the embryo into *N*_strips_ strips of equal width in the direction perpendicular to the long axis and define $\Phi_{\mathrm{dis}}(\chi )$ as the variance over *i* of the number *n*_*i*_ of particles located in strip *i* at the rescaled time *χ*. This average variance, $\left\langle \Phi_{\mathrm{dis}}(\chi )\right\rangle$, over 100 simulations, is plotted in figure 6*c* as a function of *χ* for the choices $N_{\mathrm{strips}}=100,30\;\mathrm{and}\;10$ (in all cases, we solve for the transport of *N* = 40 particles). Here also we see a clear minimum of the variance associated with optimal spreading, at χ≈196,196 and 195 μm, respectively.

#### 3.2.4. Comparison with experiments

How do these theoretical predictions compare with the value of *χ* realized in real *Drosophila* embryos? This may be estimated from the experimental results in [18].

**Figure 7.**
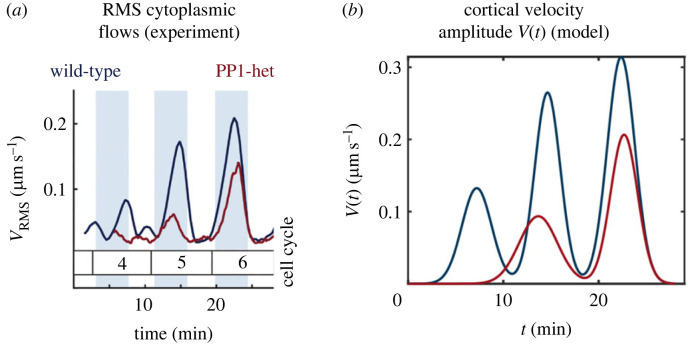
Estimation of *χ* from experiments. (*a*) RMS cytoplasmic flows measured experimentally for a wild-type (blue) and mutated (red) *Drosophila* embryo; reprinted from Deneke *et al.* [18], © (2019), with permission from Elsevier. (*b*) Model for cortical flow amplitude *V*(*t*) obtained by fitting a sum of Gaussians to the large contraction peaks in cell cycles in (*a*) and rescaling appropriately to convert RMS cytoplasmic speeds to cortical flow amplitudes.

We first estimate the cortical flow amplitude *V*(*t*) for a wild-type embryo from an experimentally measured time series of the RMS cytoplasmic flow field (reproduced in figure 7*a*, dark blue line) and the cortical velocity profile in the contraction phase of cell cycle 6 (reproduced in figure 4*a*).

We may assume that the transport is dominated by the three large cortical contraction peaks in the three cell cycles (see peaks in figure 7*a*, dark blue line), and can neglect contributions from the intervals between the peaks, since intervals with no flow lead to unchanged values of *χ*. Because our model assumes Stokes flows in the Newtonian limit, the bulk cytoplasmic flows depend linearly on the cortical flows and we may, therefore, rescale RMS cytoplasmic velocities (figure 7*a*) into cortical flow amplitudes. Therefore, we sample approximately 20 points from each contraction peak in the time series of the RMS cytoplasmic speeds (figure 7*a*, dark blue line) and rescale this data into cortical flow amplitudes, with the scaling factor chosen to obtain a cortical flow amplitude of 0.3 μm s^−1^ at the contraction peak of cell cycle 6; recall that the value *V*(*t*) = 0.3 μm s^−1^ in our model for the cortical flow, equation (3.1), is the one that gives close agreement with experiments (figure 4*c*). We then fit a sum of three Gaussians to the rescaled data to obtain a mathematical estimate as $V(t)=\Sigma_{i=1}^{3}a_{i}\exp[-{\left(t-b_{i}\right)}^{2}/2c_{i}^{2})]$ for the time-variation of the cortical flow amplitude; the resulting model for the function *V*(*t*) is illustrated in figure 7*b*.

This quantitative model for the cortical flow amplitude *V*(*t*) can then be integrated in time, leading to an estimate for the value of *χ* occurring in *Drosophila* embryos. Using a fit of three Gaussians at the 95% confidence interval level, we obtain the estimated range for *χ* of 112–147 μm, with mean value 〈*χ*〉 ≈ 129 μm. These mean values are illustrated in figure 5*b*,*c* with a black vertical line, and dotted lines are used as error bars for the range of *χ* at the 95% confidence level. Remarkably, we see from these results that the magnitude of the cortical contractions in experiments is close to the optimal value predicted theoretically by our simple mathematical model (the normalized variance corresponding to the experimental estimate of χ is only 0.07 above the theoretical optimum for *R* = 7 μm, and 0.13 higher for *R* = 28 μm). Real-life cortical flows appear thus to be near-optimal to ensure axial spreading of nuclei.

In the experiments of Deneke *et al.* [18], an embryo with mutations (named ‘PP1-heterozygous’ or ‘PP1-het’) had dampened cytoplasmic flows; this led to non-uniform nuclear spreading, and consequently, improper control and asynchrony in the following cell cycles, impairing the development process. The value of *χ* realized for this mutant can be similarly estimated by fitting a sum of two Gaussians $V(t)=\Sigma_{i=1}^{2}A_{i}\exp[-{\left(t-B_{i}\right)}^{2}/2C_{i}^{2})]$ (figure 7) to the time series of the cortical velocity amplitude rescaled from the experimentally measured RMS cytoplasmic velocity in this embryo (figure 7*a*). For this embryo, the value of *χ* realized is 〈χ〉≈ 57 μm, with ranges of error estimated to be 47–68 μm at the 95% confidence level of the fit. These values are illustrated in figure 5*b*,*c* as red solid line (mean) and dotted lines (95% error bars). We see that dampening the flows leads to a corresponding decrease in the extent of nuclear spreading achieved, captured quantitatively by a significantly smaller value of *χ* (the normalized variance corresponding to the experimental estimate of *χ* is now 0.35 above the theoretical optimum for *R* = 7 μm, and 0.48 higher for *R* = 28 μm).

#### 3.2.5. Impact of cell division

Our model of transport with passive tracers neglects the fact that the nuclei divide in each cell cycle. However, incorporating cell division and the resulting microtubule aster migration-induced nuclear separation would lead to additional spreading and homogenization, allowing the system to achieve optimal spreading at a lower value of *χ*, and thus bringing our theoretical optimum even closer to the experimental estimates.

To show this more quantitatively, we focus on the results in figure 5*d* using the kernel length scale *R* = 28 μm, since this is the nuclear separation length reported experimentally [39] and is thus the appropriate length scale to use when dividing nuclei are considered. Indeed, in order for Φ to be a measure of homogeneity that can be interpreted meaningfully across different numbers of nuclei in the embryo at different cell cycles, the single-peak density function associated with a single isolated nucleus should remain approximately the same after it divides into two (in the absence of flow). Using *R* = 28 μm ensures this, while using a significantly smaller value of *R* results in a qualitatively different two-peak density function for the daughter nuclei.

We performed simulations of cell division in the absence of flow, modelling the nuclear separation as a repulsive force field of range 28 μm around each nucleus. Specifically, 32 nuclei distributed uniformly in a spheroidal cloud of semi-major axis 140 μm and semi-minor axis 40 μm, a configuration which approximates the beginning of cell cycle 6, are allowed to divide into 64 nuclei. The values of ⟨Φ⟩ before and after cell division are computed over 100 simulations, and we find a decrease of approximately 6% in the value Φ due to one round of cell division.

We expect, however, that the decrease in ⟨Φ⟩ due to the three cell divisions occurring in cell cycles 4–6 will be less than 18% (i.e. three times 6%) of the initial value at *χ* = 0, primarily because the later cell divisions occur at values of Φ which have already been lowered by cytoplasmic flow. Furthermore, the estimate of 6% was obtained from a configuration which emulates the beginning of cell cycle 6, when the nuclei are most numerous and closely packed and when, therefore, the spreading induced by physical repulsion would be a maximum. We estimate that incorporating cell division into the full model in figure 5*c* would contribute a decrease in ⟨Φ⟩ relative to ⟨Φ(χ=0)⟩ of around 10%. The optimum value of *χ* with cell division included can, therefore, be estimated as the value of *χ* at which ⟨Φ(χ)⟩/⟨Φ(0)⟩ attains a value 0.1 higher than its minimum value (without cell division). This value is *χ* ≈ 140 μm, which is significantly closer to the experimental estimate.

## 4. Long-wavelength theory

Thus far, we have investigated flow-based transport in the *Drosophila* embryo using our analytical solution, written as an infinite series of spheroidal harmonics, for the cytoplasmic flow. This flow solution is exact, but is restricted to cell shapes that are exactly spheroidal. In this section, we use a long-wavelength assumption (also known as lubrication theory in the context of fluid mechanics) to derive a much simpler solution for the flow, which nonetheless approximates the exact flows remarkably well. Importantly, this approach is applicable to any elongated (axisymmetric) shape, and is thus not restricted to spheroidal cells.

We work in cylindrical coordinates (*r*, *ϕ*, *z*), with *z* aligned with the long axis of the cell, and denote the radius of the cell by *R*(*z*). The key assumption is that length scales in the longitudinal direction are much larger than radial length scales [40]; this means the long-wavelength model is valid in the limit |*R*′(*z*)| ≪ 1. We seek a solution for the cytoplasmic flow **u**(*r*, *z*, *t*) = *u*_*z*_(*r*, *z*, *t*)**e**_*z*_ + *u*_*r*_(*r*, *z*, *t*)**e**_*r*_. Under the long-wavelength assumption, the cell geometry is locally cylindrical, and there are no radial pressure gradients to leading order. The leading-order longitudinal flow is, therefore, pressure-driven pipe flow subject to a slip velocity *u*_*z*_ = *U*(*z*, *t*) at *r* = *R*(*z*), where

4.1 $$U(z,t)=e_{z}\cdot v_{s}{|}_{\zeta =z/b_{z}},$$

is the component along the *z*-direction of the cortical velocity **v**_*s*_. Since the ends of the cell are closed and the fluid is incompressible, there is no net mass flux through any cross-section, i.e. $\int_{0}^{R}u_{z}r\;dr=0$.

We now derive the slowly varying flow solution which satisfies these conditions. The radial component of the Stokes equations for an incompressible fluid of shear viscosity μ in the lubrication limit reads ∂*p*/∂*r* = 0, where *p* is the dynamic pressure. Note that ∂*p*/∂*θ* = 0 follows from the assumption of axisymmetry. This allows us to straightforwardly integrate the axial component of the Stokes equations in cylindrical coordinates, $\mu \nabla^{2}u_{z}=\partial p/\partial z$, to yield the general solution

4.2 $$u_{z}=\frac{1}{4\mu }\left(\frac{\partial p}{\partial z}\right)r^{2}+A\ln r+B,$$

where *A* and *B* are integration constants. Regularity of the flow at the origin requires *A* = 0. The unknown pressure gradient ∂*p*/∂*z* and the constant *B* may then be determined by imposing (i) the slip velocity boundary condition *u*_*z*_ = *U*(*z*, *t*) at *r* = *R*(*z*) and (ii) zero total mass flux throughout the cell, $\int_{0}^{R}u_{z}r\;dr=0$, yielding the final long-wavelength solution

4.3 $$u_{z}(r,z,t)=U(z,t)\left[2{\left(\frac{r}{R(z)}\right)}^{2}-1\right],$$

for the longitudinal (axial) velocity in cortically driven cytoplasmic flow. Integrating the incompressibility condition, ∇⋅u=0, for the radial velocity *u*_*r*_ then yields

4.4 $$u_{r}(r,z,t)=\frac{1}{2}\frac{\partial U(z,t)}{\partial z}r\left[1-{\left(\frac{r}{R(z)}\right)}^{2}\right]+U(z,t)\frac{dR(z)}{dz}{\left(\frac{r}{R(z)}\right)}^{3}.$$

Note that regularity at *r* = 0 required the integration constant to vanish, and the solution then satisfies the no-penetration boundary condition on the cell wall, (*u*_*z*_**e**_*z*_ + *u*_*r*_**e**_*r*_) · **n** = 0, where $n\propto (dR/dz\;e_{z}\text{–}e_{r})$ is a vector normal to the boundary. Together with the slip velocity *u*_*z*_ = *U*(*z*, *t*), this is equivalent to the original no-slip boundary condition in equation (2.2), **u** = **v**_*s*_. The long-wavelength solution in equations (4.3) and (4.4) satisfies, therefore, the boundary conditions of the exact problem.

To illustrate the prediction of this model, we reproduce in figure 3*b* the first four spheroidal modes from the exact solution in §2 but computed using the long-wavelength solution; the exact modes, determined using the spheroidal harmonics solution, are shown in figure 3*a*, and excellent agreement is seen between the two.

**Figure 8.**
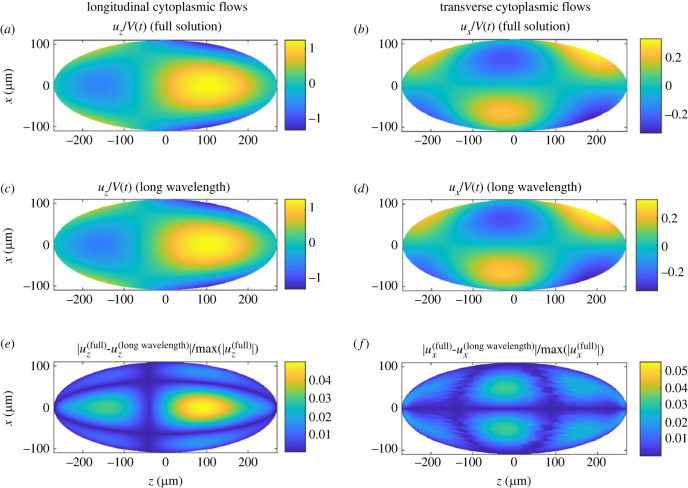
Cytoplasmic flows in the *Drosophila* embryo using two different solution methods. Longitudinal component *u*_*z*_ (*a*) and transverse component *u*_*x*_ (*b*) of the cytoplasmic flow as produced by the full spheroidal harmonics solution. Longitudinal (*c*) and transverse (*d*) components as produced by the long-wavelength solution. Errors incurred by the long-wavelength approximation for the longitudinal (*e*) and transverse (*f*) components of cytoplasmic flow relative to the full spheroidal harmonics solution, normalized by the maximum values of *u*_*z*_ and *u*_*x*_, respectively.

We can then apply our long-wavelength solution to the cytoplasmic flows in a *Drosophila* embryo, i.e. the flow driven by the slip velocity given by equation (3.1). In figure 8, we plot the longitudinal flows along the embryo axis, *u*_*z*_, and the transverse flows, *u*_*x*_, as determined by the full solution in spheroidal harmonics (figure 8*a*,*b*), compared with the long-wavelength solution (figure 8*c*,*d*). It is evident that the approximate solution is able to reproduce all features of the full solution. To quantify this further, we display the errors incurred by the long-wavelength approximation relative to the maximum velocity in the domain, $\left|u_{i}^{\mathrm{full}}-u_{i}^{\mathrm{long}\;\mathrm{wavelength}}|/\max|u_{i}^{\mathrm{full}}\right|$, in figure 8*e*,*f*. Although it is strictly only valid in the long-wavelength limit, we see that the long-wavelength solution reproduces a remarkably accurate approximation of the full solution in the entire embryo, incurring errors of less than 5%.

## 5. Reduced model of transport

In this final section, we show how to exploit the long-wavelength flow solution to derive a reduced-order model of nuclear transport. By projecting the full nuclear transport problem onto the embryo AP axis, we show that it admits an analytical solution that reproduces all the essential physics of the full system, including the characteristics of optimal transport.

### 5.1. Model formulation and solution

We first define a number density function *n*(*z*, *t*) of nuclei along the AP axis; this can be thought of as the projection of the density field along the central axis of the embryo. We then model the advection of nuclei by cytoplasmic flows as the advection of this density function by the cytoplasmic velocity, *u*_AP_(*z*, *t*), along the AP axis. In the absence of diffusion, the field *n*(*z*, *t*) is governed by the one-dimensional advection equation

5.1 $$\frac{\partial n}{\partial t}+\frac{\partial }{\partial z}(u_{\mathrm{AP}}n)=0.$$

To further simplify the problem, we shift the asymmetric cortical flows, equation (3.1), into a symmetric sine profile, preserving its amplitude, and thus take

5.2 $$v_{s}(\zeta ,t)=-V(t)\sin(\pi \zeta ).$$

This merely shifts the centre of the advective field but does not modify the essential features of the transport problem.

From our lubrication solution, the cytoplasmic velocity *u*_AP_(*z*, *t*) along the AP axis may be expressed in terms of the cortical flow *v*_*s*_(*ζ*, *t*) as

5.3 $$u_{\mathrm{AP}}(z,t)=-v_{s}\left(\frac{z}{b_{z}},t\right)e_{\zeta }\cdot e_{z}.$$

In the bulk, $e_{\zeta }\cdot e_{z}\approx 1$ because the cortex is approximately parallel to the AP axis; near the poles, where $e_{\zeta }\cdot e_{z}$ deviates significantly from unity, *v*_*s*_ is small and therefore the absolute difference between the component of **v**_*s*_ parallel to the cortex and the component parallel to the AP axis remains small. Therefore, it is consistent to make the approximation *u*_AP_(*z*, *t*) = −*v*_*s*_(*z*/*b*_*z*_, *t*) throughout. Defining the non-dimensionalized variable *Z* : = *z*/*b*_*z*_, we may, therefore, write

5.4 $$u_{\mathrm{AP}}(Z,t)=V(t)\sin(\pi Z).$$

Finally, we define a rescaled time

5.5 $$T\;:=\frac{\pi }{b_{z}}\int_{0}^{t}V(t^{\prime })\;dt^{\prime }=\frac{\pi }{b_{z}}\chi ,$$

so that, using these new variables, the transport equation, equation (5.1), simplifies to

5.6 $$\frac{\partial n}{\partial T}+\frac{\partial }{\partial Z}\left[\frac{\sin(\pi Z)}{\pi }n\right]=0.$$

Starting from the initial condition for the nuclei density *n*(*Z*, 0) = *n*_0_(*Z*), we can use the method of characteristics to obtain the exact time-dependent solution as

5.7 $$n(Z,T)=e^{-T}n_{0}\left(\frac{2}{\pi }\arctan\left(e^{-T}\tan\frac{\pi Z}{2}\right)\right)\frac{\tan^{2}(\pi Z/2)+1}{e^{-2T}\tan^{2}(\pi Z/2)+1}\cdot$$

### 5.2. Evolution of nuclear density and optimal axial spreading

In order to facilitate comparison between the full simulations (§3) and the reduced model (§5.1), we revert back to the rescaled time variable, *χ* = *Tb*_*z*_/*π*. We, therefore, now consider the density function *n*(*Z*, *χ*) as a function of the variables *Z* and *χ*.

To show how the reduced model captures the essential features of the problem, we consider how a cloud of nuclei centred at the origin is transported by it. We mimic the initial conditions used in the full simulations, i.e. a uniform distribution of nuclei in a sphere of radius 60 μm, and thus make the specific choice $n(Z,0)=n_{0}(Z)=\sqrt{\max(0,Z_{0}^{2}-Z^{2})}/(\pi Z_{0}^{2}/2)$ with *Z*_0_ = (60 μm)/*b*_*z*_.

**Figure 9.**
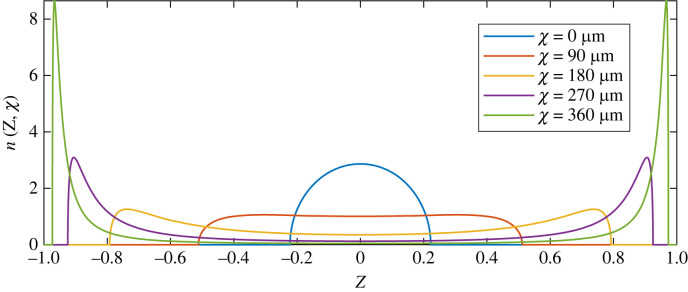
Nuclear transport by the reduced model. Profiles of *n*(*Z*, *χ*) for increasing values of *χ*, to be compared with figure 5*a* (same values of *χ*). The variance of *n*, a measure of axial spreading, is plotted as a function of *χ* in figure 5*b* (purple dashed line).

We plot in figure 9 the profiles of *n*(*Z*, *χ*) for five values of the rescaled time *χ*. Similarly to what was seen in the full simulations (figure 5*a*, same values of *χ*), the cytoplasmic flows initially spread the cloud of nuclei along the cell axis, but beyond some value of *χ* applying further flows hinders axial homogenization and instead creates a ‘neck’ scarce in nuclei in the centre of the cell.

Similarly to our analysis of the full simulations, we may further quantify the nuclear spreading along the cell axis by defining a measure of axial homogeneity for the reduced one-dimensional model. We compute the variance, $\Phi_{1D}(\chi )$, of *n*(*χ*, *Z*) over the full spatial interval *Z* ∈ [−1, 1]

5.8 $$\Phi_{1D}(\chi )\;:=\int_{-1}^{1}{\left[n(\chi ,Z)-\bar{n}\right]}^{2}\;dZ,$$

where $\bar{n}\equiv 1/2$ is the nuclear density averaged over *Z* (note that $\bar{n}$ is independent of *χ* since nuclei are conserved; this may be seen directly by integrating equation (5.6) over *Z*). This variance is plotted against *χ* in figure 5*b* (purple dashed line). Remarkably, despite the drastic simplifications in the one-dimensional approach, the reduced model is able to capture all essential physics of nuclear transport, with axial spreading that is poor at low *χ* (nuclei initially concentrated in the centre of the cell) and high *χ* (nuclei accumulating at the poles; note that the variance in the reduced model overshoots that in the full simulations at large *χ* because recirculation effects cannot be accounted for in one dimension). Therefore, here again, axial nuclear transport is optimal for a finite value of *χ*; the variance in the reduced model is minimized at *χ* ≈ 159 μm (purple diamond in figure 5*b*), which falls in between the predicted optimal values *χ* ≈ 195 and 213 μm obtained in the full model with *R* = 7 and 28 μm, respectively (§3.2.3), and the empirical value *χ* ≈ 129 μm estimated from the experiments (§3.2.4).

## 6. Discussion and conclusion

In this paper, motivated by cortex-driven flow-based transport of nuclei in the embryo of the model organism *D. melanogaster*, we proposed two mathematical models to study cytoplasmic streaming and boundary-driven flows inside elongated biological cells. Assuming (i) that the cytoplasm behaves like a Newtonian fluid at low Reynolds number, (ii) that the cell has a prolate spheroidal shape, and (iii) that the flow is driven by a prescribed axisymmetric tangential velocity on the boundary of the cell, we first computed the entire flow field analytically.

To demonstrate the usefulness of such an analytical modelling approach, we then considered recent experiments characterizing the transport of nuclei in cell cycles 4–6 of the *Drosophila* embryo. By fitting the cortical contractions in our theoretical model to experimental data, and introducing two different measures of axial spreading of the nuclei along the long axis of the embryo, we revealed that experimental cortical flows ensure near-optimal spreading of the nuclei along the embryo.

We next further simplified our theoretical approach and derive a second model for the flow under the long-wavelength (lubrication) approximation. This approach provides a simpler solution for the flow—and therefore a more practical one—which reproduces the exact solution with errors of 5% or less without the need to evaluate integrals of the boundary flow (equation (2.23)). We then used our long-wavelength solution in a reduced continuum model for nuclear transport, leading to analytical solutions for the nuclear concentration that capture the essential physics of the full system, including optimal axial spreading.

Obviously, the modelling approach in this paper is focused on flow transport and thus neglects some of the physico-chemical parameters that may play key roles in cortical dynamics. We have also modelled the system as a single Newtonian fluid driven by slip velocities at the boundary, and thus neglected possible complex rheological properties of the cytoplasm and cortex. This is in contrast to the recently introduced, more detailed model of the same system as an active actomyosin gel coupled to a passive viscous cytoplasm [25]; this two-fluid model can be solved numerically to quantitatively reproduce the detailed features of the experimental flow field, and in particular its deviations from a Stokes flow. However, a simple Stokes flow model as is used here does successfully explain the large-scale features of the flow and transport and in turn, these simplifications enable us to bypass the need for numerical computations in determining the flows and thus can be exploited to gain fundamental biophysical insight on the impact of cytoplasmic flows on cellular transport. The model is versatile and may be adapted to the case of other cell shapes, or coupled with more complex models of the active cortex, for example, one that includes detailed biochemical feedback between cortical flows and nuclear positioning [25].

## Data Availability

Relevant code is available from the Zenodo repository: https://doi.org/10.5281/zenodo.8398819 [41].
